# Inactivation of the Pyrimidine Biosynthesis *pyrD* Gene Negatively Affects Biofilm Formation and Virulence Determinants in the Crohn’s Disease-Associated Adherent Invasive *Escherichia coli* LF82 Strain

**DOI:** 10.3390/microorganisms10030537

**Published:** 2022-02-28

**Authors:** Elio Rossi, Gabriella Leccese, Valerio Baldelli, Alessia Bibi, Emanuele Scalone, Carlo Camilloni, Moira Paroni, Paolo Landini

**Affiliations:** Department of Biosciences, Università degli Studi di Milano, 20133 Milano, Italy; elio.rossi@unimi.it (E.R.); gabriella.leccese@unimi.it (G.L.); valerio.baldelli@unimi.it (V.B.); alessia.bibi@unimi.it (A.B.); emanuele.scalone@unimi.it (E.S.); carlo.camilloni@unimi.it (C.C.)

**Keywords:** adherent-invasive *E. coli* (AIEC), Crohn’s disease, dihydroorotate dehydrogenase (DHOD), curli fibers, stress response, virulence, adhesion factors

## Abstract

In Crohn’s disease (CD) patients, the adherent-invasive *Escherichia coli* (AIEC) pathovar contributes to the chronic inflammation typical of the disease via its ability to invade gut epithelial cells and to survive in macrophages. We show that, in the AIEC strain LF82, inactivation of the *pyrD* gene, encoding dihydroorotate dehydrogenase (DHOD), an enzyme of the de novo pyrimidine biosynthetic pathway, completely abolished its ability of to grow in a macrophage environment-mimicking culture medium. In addition, *pyrD* inactivation reduced flagellar motility and strongly affected biofilm formation by downregulating transcription of both type 1 fimbriae and curli subunit genes. Thus, the *pyrD* gene appears to be essential for several cellular processes involved in AIEC virulence. Interestingly, vidofludimus (VF), a DHOD inhibitor, has been proposed as an effective drug in CD treatment. Despite displaying a potentially similar binding mode for both human and *E. coli* DHOD in computational molecular docking experiments, VF showed no activity on either growth or virulence-related processes in LF82. Altogether, our results suggest that the crucial role played by the *pyrD* gene in AIEC virulence, and the presence of structural differences between *E. coli* and human DHOD allowing for the design of specific inhibitors, make *E. coli* DHOD a promising target for therapeutical strategies aiming at counteracting chronic inflammation in CD by acting selectively on its bacterial triggers.

## 1. Introduction

Crohn’s disease (CD) is characterized by chronic intestinal inflammation resulting from inappropriate and persistent activation of the intestinal mucosal immune system [1]. The pathophysiology of CD is multifactorial, including genetic and environmental factors resulting in an aberrant immune response [2,3,4,5,6]. Arguably, however, one of the main factors in CD pathogenesis is gut microbiota dysbiosis [7,8]. Indeed, metagenomic analysis of human gut microbiota in CD patients has outlined clear pattern changes in microbial abundances in comparison to healthy individuals, such as a depletion of symbionts belonging to the Firmicutes phylum, like Bifidobacteria and Clostridia [9,10]. It is thought that these bacterial species might exert a protective effect by production of short chain fatty acids, such as butyrate, with anti-inflammatory effects [11]. In contrast, adherent-invasive *Escherichia coli* (AIEC) have consistently been found to be enriched in ileal specimens from CD patients in comparison to healthy subjects [12]. Although early reports suggested a more predominant role of AIEC in CD than in ulcerative colitis (UC), the other main inflammatory bowel disease, other works point to a role of the pathogen even in the latter disease [13,14]. The important role played by AIEC in IBD pathogenicity depends on their ability to invade intestinal epithelial cells and increase their permeability, triggering secretion of pro-inflammatory cytokines, thus ultimately resulting in chronic inflammation [15,16]. In addition, AIEC strains are able to survive and even replicate within human macrophages [17], leading to over-induction of the innate immune response. An extensive genetic and functional analysis, mostly carried out on the AIEC strain LF82, has identified several virulence factors and regulatory pathways involved in cell invasion and the induction of chronic inflammation in the human host, namely adhesion factors such as type 1 pili [18,19], specific invasive genes such as *ibeA* [20], regulatory genes such as the σ^E^ network [21], and efflux pumps [22]. 

Due to the direct role played by microorganisms in the disease, antibiotic treatments are routinely used in Crohn’s disease treatment: for instance, an association of metronidazole and of the anti-inflammatory drug azathioprine has been shown to be effective in preventive post-operative Crohn’s recurrence [23]. Interestingly, azathioprine is itself an antimicrobial drug, able to inhibit growth of *Mycobacterium avium* ssp. *paratuberculosis*, another bacterium associated with Crohn’s disease [24], and to impair virulence factors’ production in AIEC [25]. However, the long-term effectiveness of antibiotic treatments is questionable, as they seem to further promote dysbiosis, thus not tackling, or sometimes even exacerbating, one of the main triggers for chronic inflammation [26]. To ameliorate dysbiosis, probiotics, especially bacteria of the Lactobacillus and Bifidobacterium genera, have been utilised on CD patients, but clinical studies on probiotic efficacy are inconclusive [27,28,29]. Recently, we have performed an in vitro study comparing the immunomodulatory effects of Lactobacilli and Bifidobacteria probiotic strains, showing that production of pro-inflammatory cytokines and the activation of the IL-23/Th17 axis in response to AIEC is effectively counteracted by probiotics in cells from healthy subjects and from individuals suffering with ulcerative colitis, but not in cells from CD patients [30], thus suggesting that probiotics might have a limited impact in CD.

In this manuscript, we report that a mutation inactivating the *pyrD* gene of the AIEC LF82 strain results in the loss of its ability to grow in a macrophage environment-mimicking medium and inhibits biofilm and virulence determinants, such as curli fibers, type 1 fimbriae, and flagellar motility. The *pyrD* gene encodes dihydroorotate dehydrogenase (DHOD), an enzyme involved in the de novo biosynthesis of pyrimidines. Thus, our results point to a role of intracellular pyrimidine concentrations as a regulatory signal for genes involved in virulence and in host interaction, as already identified for other pathogenic bacteria such as *Pseudomonas aeruginosa* and *Shigella flexneri* [31,32]. Based on the observation that *E. coli* DHOD not only differs from the human enzyme, but also has a low sequence identity to DHOD from probiotic species like *Lactococcus lactis*, we propose that the development of specific inhibitors of *E. coli* DHOD might be a potentially interesting therapeutic strategy for CD remission via selective inhibition of AIEC growth and virulence. 

## 2. Materials and Methods

### 2.1. Transposon Mutagenesis, Mutant Identification, and Screening on Acidic and Nutrient-Poor Medium

Transposon insertion mutagenesis was carried out using the EZ-Tn5<R6Kγori/KAN-2> transposome (Lucigen, Middleton, WI, USA) on LF82, an AIEC strain originally isolated from an ileal biopsy specimen from a CD patient [33]. Transposon mutagenesis and the determination of a transposon insertion site by rescue cloning were carried out according to the manufacturer’s instructions. Through multiple transformation rounds, we obtained a library of 10,058 kanamycin resistant mutants, i.e., a 2.2× coverage of the *E. coli* LF82 genome (4534 genes). Growth in acidic and nutrient-poor medium (Acid Medium: 100 mM bis-Tris, 0.1% Casamino Acids, 0.16% glycerol, and 10 µM MgCl_2_, and the pH was adjusted to 5.8 with 10M HCl) [34] was determined as follows: overnight bacterial cultures grown in Yeast extract/Casamino acid (YESCA) medium (10 g/L casamino acids, 1.5 g/L Yeast extract) at 37 °C were diluted to OD_600_ = 0.02 in Acid Medium and overnight growth was constantly monitored in a microplate reader (SAFAS MP96). 

### 2.2. Adhesion Factor Detection, Biofilm Quantification, Motility Assay, MIC Determination and LPS Integrity Evaluation

For phenotypical assays, bacteria were grown in YESCA medium, either at 30 °C or at 37 °C. When necessary, uracil was added at 0.25 mM from a 50 mM uracil solution in 50% dimethyl sulfoxide (DMSO); 0.25% DMSO was always added to control cultures. For adhesion factor detection, bacteria were grown on YESCA agar medium supplemented with either 0.004% Congo red and 0.002% Coomassie blue (CR) or 0.005% calcofluor (CF); dyes were always added to the medium after autoclaving. Bacteria were grown for 24 h at either 30 °C or 37 °C; phenotypes were better detected after a further 24–48 h incubation at 4 °C. 

Biofilm formation was determined using the crystal violet (CV) assay as described previously [25]. For flagellar motility assays, bacterial cells were grown overnight in YESCA and normalized to an OD_600_ = 1. For each culture, 3 µL were spotted at the centre of a motility agar plate in the same growth medium used for overnight cultures, supplemented with 0.3% agar. Motility was determined by the diameter of the area colonized by the bacteria after 15 h of growth at 37 °C. 

Determination of minimal inhibitory concentrations (MIC) of vidofludimus for LF82 was performed using standard 1:2 dilution methods in liquid YESCA medium, using an inoculum of 2 × 10^5^ cfu/mL. To assess vidofludimus antimicrobial activity on Gram positive bacteria, we performed MIC determination on *Bacillus cereus* strain 971 and *Staphylococcus epidermidis* ATCC 155.

To evaluate LPS integrity, overnight cultures of LF82 and LF82*pyrD::*Tn5 mutants grown in YESCA medium were normalized to OD_600_ = 1.0 and serially diluted 1:10 six times. 3 µL of each dilution were spotted on LB and MacConkey agar in triplicates and plates were incubated overnight at 37 °C.

### 2.3. Gene Expression Determination by Quantitative Real-Time PCR

For RNA isolation, strains were grown either to mid-log phase or to the onset of the stationary phase in YESCA medium at 30 °C. Bacterial cells were harvested by centrifugation at 10,000× *g* for 5 min at 4 °C and cell pellets resuspended in 300 µL of DNA/RNA Shield (Zymo Research, Irvine, CA, USA). Total RNA was extracted using the Quick-RNA Miniprep Kit (Zymo Research, Irvine, CA, USA) after the addition of 1 mg/mL Lysozyme and 400 µg/mL Proteinase K to the ruspended pellet. RNA samples were checked by agarose gel electrophoresis to assess lack of degradation and quantified spectrophotometrically. Genomic DNA removal and reverse transcription were performed on 1 μg of total RNA, along with negative control samples incubated without reverse transcriptase using the QuantiTect Reverse Transcription Kit (Qiagen, Germantown, MD, USA). cDNA synthesis efficiency was verified by electrophoresis on agarose gel in comparison to negative controls. Real-time PCR was performed using the SYBR Green PCR master mixture, and the results were determined with a Rotor-Gene 3000 detection system (Corbett Research, Saffron Walden, UK). Reaction mixtures (15 μL) included 0.1 μg cDNA and 300 nM primers in the reaction buffer and enzyme supplied by the manufacturer. Primer sequences are listed in Table S1. A minimum of three biologically independent experiments were considered for analysis; negative control samples (i.e., non-retrotranscribed RNA) never showed significant threshold cycles. The relative transcript amounts were determined using 16S rRNA as the reference gene ([CtGene of interest-Ct16S] = ΔCt value).

### 2.4. Computational Models for Vidofludimus Binding to Dihydroorotate Dehydrogenase (DHOD) from Different Organisms

Docking calculations were performed using Glide version 2021.3. Receptor structures (hDHOD, PDB code: 2PRL and EcDHOD, PDB code: 1F76) were prepared using the Protein Preparation Wizard of the Maestro graphical user interface (Schrödinger suite https://www.schrodinger.com/, Schrödinger, Mannheim, Germany) by removing all crystallographic waters and additives and optimizing the orientation of hydrogen bonds and the protonation state of histidine, aspartic acid and glutamic acid. The protein preparation was followed by a restrained minimization of the whole system. Target grids were built on receptor structures. Ligand sampling was set to ’Flexible’ and we included the Epik state penalties in the docking score. For each compound, 5 poses were saved after a post-minimization of the ligand structure within the binding site. The docking protocol was initially tested for its ability to reproduce the binding mode of the native R2C in the crystal structure (2PRL). The program was successful in reproducing the experimentally determined binding mode of the compound as it corresponds to the best-scored pose. Then, docking with VF was followed by a 50 ns long Molecular Dynamics (MD) simulation using the first predicted pose. MD simulations were performed using the Desmond package of the Schrödinger suite. The chosen force field was OPLS4. The systems were solvated in a dodecahedron hexagon box with a 10 Å buffer and using TIP3P as water and 0.15 M of NaCl. MD simulation was preceded by energy minimization and equilibration steps.

### 2.5. Statistical Analysis

Statistical analysis was performed with Prism 9 software (GraphPad Software, San Diego, CA, USA). Student’s *t*-tests for unpaired or paired samples were used to evaluate differences between means. Statistical significance between the means of more than two groups were performed using one-way ANOVA computing the Tukey’s multiple comparisons test to evaluate differences between groups.

## 3. Results

### 3.1. Mutant Selection in an Acidic and Nutrient Stress Medium Mimicking the Macrophage Vacuole Environment

**Figure 1:**
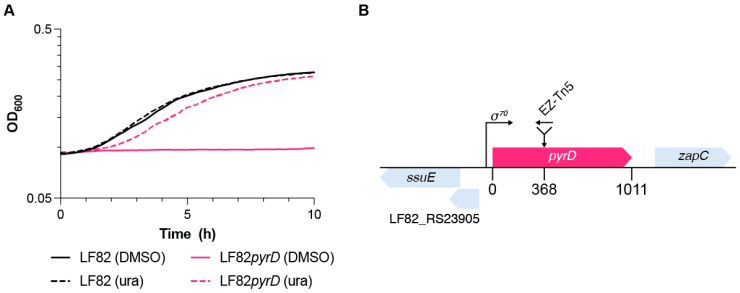
(**A**) Growth curves for LF82 (black) and LF82*pyrD::*Tn5 (LF82*pyrD* magenta) in acid medium mimicking the macrophage environment, either in the presence of 0.25 mM uracil (dashed lines) or 0.25% dimethyl sulfoxide DMSO (solid lines); (**B**) Localization of the EZ-Tn5<R6Kγori/KAN-2> transposon insertion site in the *pyrD* gene.

In order to identify genes that might be involved in AIEC virulence, we created a transposon mutagenesis library in the LF82 strain, using the EZ-Tn5<R6Kγori/KAN-2> transposome (Lucigen, WI, USA). Transposon insertion mutants were screened for their ability to grow in acid medium, recreating the harsh environment of the macrophage vacuole, characterized by low pH and limited nutrient availability [34]. Indeed, in this medium, even the LF82 parental strain was only able to carry out roughly two replications, with a maximal growth rate of 0.24 h^−1^ (Figure 1A and Figure S1). Out of the 10058 transposon mutants screened, 141 showed either a complete loss or a strong reduction in their ability to grow in acid medium, which was, however, often accompanied by growth defects also in the YESCA medium. Thus, we focused our attention on one mutant whose growth was not affected in YESCA, but totally abolished in acid medium. This mutant was compared to its parental LF82 strain in a more standardized experiment in which fresh overnight cultures of either strain grown in YESCA were resuspended to an OD_600_ = 0.085 and incubated overnight in acid medium, confirming total lack of growth by the mutant (Figure 1A). 

The transposon insertion was mapped within the *pyrD* gene, encoding DHOD, the enzyme catalyzing the fourth step of the de novo pyrimidine biosynthesis, and the mutant strain will be referred to as LF82*pyrD::*Tn5 from now on. The transposon insertion site lies at nucleotide 368 of the 1011-bp long *pyrD* gene, immediately downstream of a portion of the gene coding for a domain involved in substrate binding, thus suggesting functional inactivation of the *pyrD* gene (Figure 1B). Loss of *pyrD* function was confirmed by restoration of LF82*pyrD::*Tn5 growth in acid medium by addition of 0.25 mM uracil (Figure 1A), which would also suggest that absence of exogenous pyrimidines, rather than acid sensitivity, is the reason for LF82*pyrD::*Tn5 inability to grow in this medium. Indeed, AIEC mutants deficient in pyrimidine biosynthesis have already been shown to be unable to survive in macrophages [35]. Although exogenous uracil rescued its defective phenotype in acid medium, the LF82*pyrD::*Tn5 mutant still showed a longer lag phase (120 vs. 75 min) and a slightly, albeit statistically significant, slower growth rate (0.21 vs. 0.24 h^−1^) than its parental strain even when exogenous uracil was provided (Figure 1A and Figure S1), suggesting that the *pyrD* mutation might affect LF82 fitness in acid medium even in the presence of excess pyrimidine availability.

### 3.2. The LF82pyrD::Tn5 Mutant Is Impaired in Biofilm Formation and Adhesion Factors’ Production and Displays a Slightly Reduced Flagellar Motility

In previous works, we showed that perturbations of intracellular nucleotide pools strongly affect biofilm formation and adhesion factors’ production in *E. coli*: indeed, in the *E. coli* MG1655 laboratory strain, mutations in de novo pyrimidine biosynthesis genes strongly downregulate curli production, a major adhesion factor [36]. Likewise, the purine analogue azathioprine, and other 6-mercaptopurine drugs, can impair both curli fibers’ production and cell motility in LF82 [25]. 

**Figure 2:**
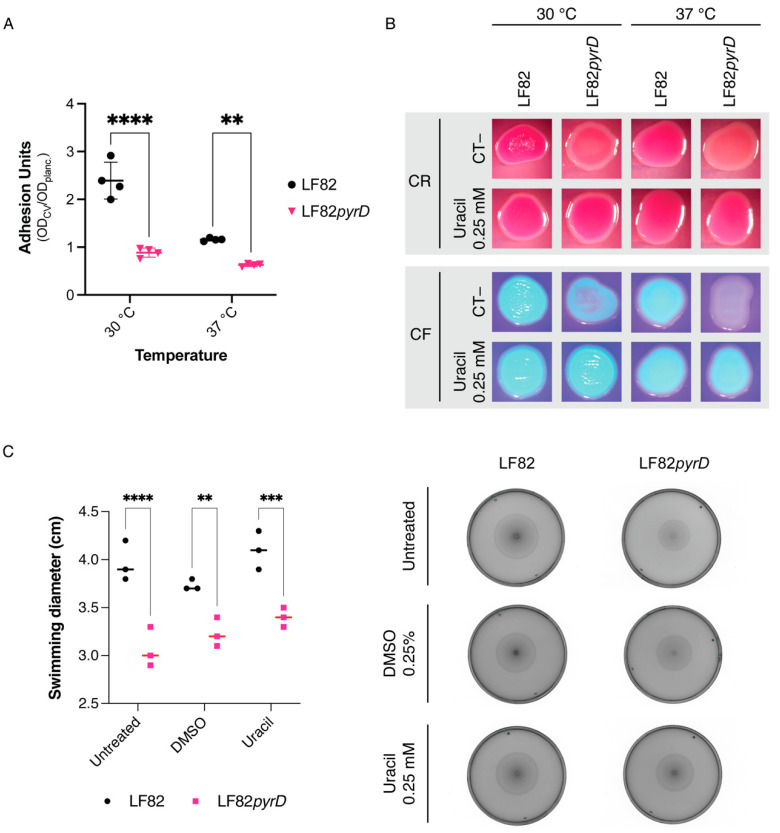
(**A**) Biofilm formation, measured as surface adhesion to polystyrene microtiter plates, using the Crystal violet method. Cultures were grown overnight in Yeast extract/Casamino acid (YESCA) medium at either 30 °C or 37 °C. Results of four independent values and median are shown. **, *p*-value < 0.01; ****, *p*-value < 0.0001, One-way ANOVA with Tukey’s test for multiple comparisons; (**B**) LF82 and LF82*pyrD::*Tn5 (LF82*pyrD*) phenotypes grown either at 30 °C or 37 °C on YESCA medium, either with or without uracil supplementation, in the presence of either Congo red (CR) or Calcofluor (CF); (**C**) Swimming motility on YESCA soft agar plates (typical experiment, right) and measurement of halo diameters (three independent experiments and median are shown, left). **, *p*-value < 0.01, ***, *p*-value < 0.001, ****, *p*-value < 0.0001, Two-way ANOVA with Šidák’s test for multiple comparisons.

To assess whether the *pyrD* mutation could also impair bacterial adhesion and cell motility, both involved in AIEC virulence, we performed crystal violet biofilm staining and swimming motility assays comparing LF82 to its LF82*pyrD::*Tn5 derivative (Figure 2A). Since curli fibers production in most *E. coli* strains, including LF82 [25], is strongly inhibited at 37 °C in liquid media, we also performed biofilm and adhesion factor assays at 30 °C. Indeed, the LF82 wild type strain was more proficient in biofilm formation at 30 °C than at 37 °C, consistent with the production of curli fibers at lower growth temperatures (Figure 2A); however, the LF82*pyrD::*Tn5 mutant strain displayed a significant reduction in biofilm formation at both 30 °C and 37 °C, possibly suggesting the inhibition of multiple adhesion factors by the *pyrD* mutation (Figure 2A). The impaired ability to form biofilm by the LF82*pyrD::*Tn5 mutant is not due to any reduction in overall growth in the YESCA medium (Figure S2).

To further confirm the effects of the LF82*pyrD::*Tn5 mutation on curli fibers production, we performed phenotypic assays on solid medium supplemented with either Congo red or Calcofluor, dyes that can bind both curli fibers and the extracellular polysaccharide cellulose, often co-produced with curli, as well as other cell surface structures. While LF82 showed, respectively, red and fluorescent phenotypes on either Congo red (CR)- or calcofluor (CF)-supplemented media, binding to either dye was affected in the LF82*pyrD::*Tn5 mutant, particularly at 37 °C (Figure 2B), at which the mutant strain totally loses fluorescence on CF-supplemented medium and the pinkish coloration on CR displayed by its parental strain. Supplementation of 0.25 mM uracil fully overcame the effects of the *pyrD* mutations, restoring the ability of LF82*pyrD::*Tn5 to bind either dye (Figure 2B), thus strongly suggesting that LF82*pyrD::*Tn5 phenotypes are indeed due to a reduction of the pyrimidine availability. 

Finally, LF82*pyrD::*Tn5 is also impaired in cellular motility (Figure 2C), suggesting that perturbation of intracellular pyrimidine nucleotide pools also affects this important virulence-related cell process in AIEC [21]. Interestingly, however, unlike for CR and CF phenotypes (Figure 2B), uracil supplementation did not restore full cellular motility to the mutant strain, thus suggesting that the modulation of flagellar motility might depend on the relative concentrations of some de novo pyrimidine biosynthesis pathway intermediates rather than the overall pyrimidine intracellular concentration.

Results in Figure 2 show that the LF82*pyrD::*Tn5 strain is hindered in production of adhesion factors, in biofilm formation and in cell motility. We reasoned that mutations in de novo pyrimidine biosynthesis might reduce the pool of UTP available for the activation of sugar precursors, thus affecting the production of extracellular polysaccharides such as peptidoglycan and lipopolysaccharide (LPS). In particular, mutations affecting LPS integrity have been shown to affect both adhesion to solid surfaces and cell motility in *E. coli* [37]. Thus, in order to verify whether the LPS structure might be impaired in the LF82*pyrD::*Tn5 strain, we determined its viability on MacConkey medium, as production of incomplete or aberrant LPS makes *E. coli* sensitive to bile salts present in this medium [38]. As shown in Figure S3A, we could not detect any reduction in viability comparing LF82*pyrD::*Tn5 strain growth in LB vs. MacConkey medium, thus suggesting that inactivation of the *pyrD* gene does not result in extensive perturbation of the LPS structure. As further assessment of the integrity of the cell envelope in the LF82*pyrD::*Tn5 mutant strain, we tested the activation of the σ^E^-dependent *lptD* gene [39], as the σ^E^ regulon is activated in response to changes in LPS structure [40]: again, no induction of *lptD* transcription was observed in the *pyrD* mutant strain (Figure S3B).

### 3.3. The pyrD::Tn5 Mutation Results in Transcription Downregulation of Genes Encoding Curli Fibers and Type 1 Fimbriae

**Figure 3:**
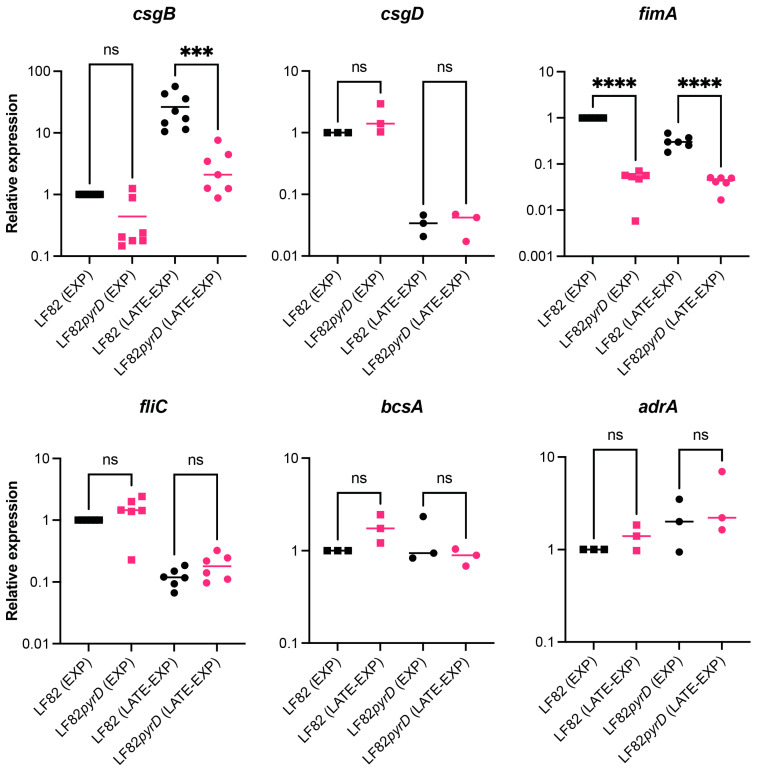
Determination of gene expression levels in the LF82 vs. the LF82*pyrD::*Tn5 (LF82*pyrD*) strain by qRT-PCR. RNA was extracted from bacterial cultures grown in YESCA medium at 30 °C either during exponential growth (EXP, OD_600nm_ = 0.4) or transition to stationary phase (LATE-EXP, OD_600nm_ = 1.0). Values are expressed as arbitrary units; transcription levels in the LF82 strain during exponential growth are set to 1. Data are from at least three independent experiments and median are shown. ns, not significant; ***, *p*-value < 0.001, ****, *p*-value < 0.0001 One-way ANOVA with Tukey’s test for multiple comparisons.

Inhibition of curli production in the LF82*pyrD::*Tn5 mutant would be consistent with our previous observations that inactivation of genes of the de novo pyrimidine biosynthetic pathway negatively impacts curli and cellulose production in the *E. coli* MG1655 laboratory strain via the downregulation of the *csgDEFG* operon [36]. The first gene of the *csgDEFG* operon codes for the CsgD regulatory protein that activates transcription of the *csgBAC* operon, encoding curli structural subunits, and of the *adrA* (*dgcC*) gene, which in turn promotes cellulose production by acting on the product of the cellulose biosynthetic *bcs* operon at the enzymatic level [41,42]. To verify this hypothesis, we compared expression of curli- and cellulose-related genes in LF82*pyrD::*Tn5 versus its parental strain. Gene expression was determined at 30 °C, both during exponential growth and at the onset of the stationary phase (Figure 3).

Inactivation of the *pyrD* gene strongly affected *csgB* expression, downregulating it by more than 10-fold, particularly at the onset of the stationary phase, in which *csgB* expression increases by 26-fold compared to the exponential phase (Figure 3). In contrast, it did not significantly affect *csgD* transcription levels, which, in contrast, were higher during the exponential phase, showing a ca. 33-fold decrease at the onset of the stationary phase (Figure 3). Our results suggest that in AIEC, unlike the *E. coli* MG1655 laboratory strain, perturbation of intracellular pyrimidine pools due to *pyrD* inactivation specifically targets *csgBAC*, encoding curli subunits, without affecting the CsgD regulon at large. Indeed, transcription of the cellulose-related *adrA* and *bscA* genes were not significantly different in LF82*pyrD::*Tn5 compared to its parental strain (Figure 3, ca. 1.3 fold WT vs. mutant). However, it must be pointed out that *adrA* transcription levels were almost undetectable also in the LF82 parental strain, possibly suggesting that *adrA* might not be transcribed at significant levels in AIEC, at least in our experimental conditions.

In addition to the curli structural operon, the *pyrD::*Tn5 mutation results in strong downregulation (ca. 20-fold in exponential phase, Figure 3) of the *fimA* gene, encoding the major subunit of type 1 fimbriae, an important virulence factor in AIEC, promoting its adhesion to epithelial cells [18,19], thus suggestted that perturbation of pyrimidine nucleotide pools negatively affects multiple adhesion factors in LF82. In contrast, *fliC*, encoding the main flagellar subunit, was not differently expressed in the LF82*pyrD::*Tn5 mutant, suggesting that reduced motility in this strain (Figure 2C) might be mediated at flagellar motility rather than at the flagellar gene transcription level.

### 3.4. Dihydroorotate Dehydrogenase (DHOD) as Potential Drug Target

Results presented so far suggest that inactivation of the *pyrD* gene, resulting in loss of dihydroorotate dehydrogenase (DHOD) activity, and consequent perturbation of intracellular pyrimidine pools, affects a variety of cellular processes involved in AIEC host colonization and virulence, such as cell adhesion and motility (Figure 2), and ability to grow in environments devoid of exogenous pyrimidines (Figure 1), such as the vacuoles formed during macrophage infection by AIEC [35]. Interestingly, inhibitors of the human DHOD protein, like vidofludimus (VF), have been widely studied as anti-inflammatory drugs in several pathologies, including CD [43]. We recently showed that purine synthesis inhibitors 6-mercaptopurines, a class of widely used anti-inflammatory drugs, possess antimicrobial activity against LF82 and can inhibit biofilm formation and motility in this bacterium at subinhibitory concentrations for growth [25], thus suggesting that their antimicrobial activity might contribute to their effectiveness in Crohn’s disease treatment. We hypothesized that VF might also inhibit *E. coli* DHOD, thus mimicking the effects of the *pyrD* mutation and hampering AIEC virulence, and that such inhibition might contribute to its anti-inflammatory action.

**Figure 4:**
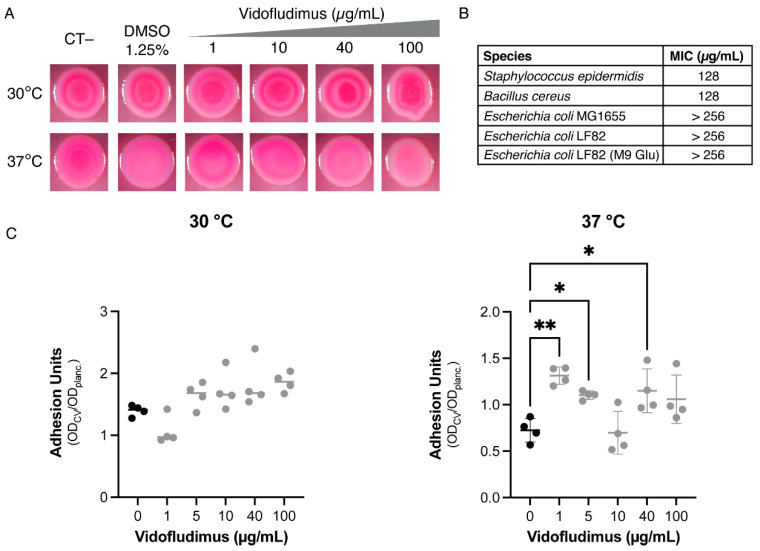
Effects of vidofludimus (VF) on LF82 phenotypes and on microbial growth. (**A**) Phenotypes on Congo red-supplemented YESCA agar medium, either at 30 °C or 37 °C, in the presence of growing VF concentrations. The final DMSO concentration was kept at 1.25% for all VF concentrations tested. (**B**) Minimal inhibitory concentrations (MIC) on *E. coli* (LF82, in YESCA and M9 Glu 0.2% media, and the laboratory strain MG1655, in YESCA medium) and the Gram positive bacteria *Bacillus cereus* and *Staphylococcus epidermidis* (in YESCA medium). (**C**) Biofilm formation, measured as surface adhesion to polystyrene microtiter plates, using the Crystal violet method, in the presence of increasing VF concentrations. Cultures were grown overnight in YESCA medium at either 30 °C or 37 °C. *, *p*-value < 0.05, **, *p*-value < 0.01 One-way ANOVA with Tukey’s test for multiple comparisons. Only significant values are shown.

Unlike 6-mercaptopurines, however, VF showed no antibacterial activity on LF82 up to 256 µg/mL (Figure 4B), and it failed to inhibit biofilm formation at either 30 °C or 37 °C (Figure 4C), while only promoting a very slight phenotypic change of LF82 on CR-supplemented media at 37 °C (Figure 4A). VF even induced a slight increase in biofilm formation that was only statistically significant at 37 °C and did not show a clear dose-dependence, suggesting that these effects are not mediated by inhibition of DHOD activity. The lack of significant biological effects on LF82 by VF might be due either to inability to enter the bacterial cells of Gram negative bacteria, despite its small molecular size (molecular mass = 355.12), or to low binding affinity to bacterial DHOD. VF showed very poor antimicrobial activity on the Gram positive bacteria *Bacillus cereus* and *Staphylococcus epidermidis* (MIC = 128 µg/mL for either species, Figure 4B), which are typically more sensitive to antimicrobial agents not able to cross the outer membrane of Gram negative bacteria, possibly suggesting poor inhibition of bacterial DHOD. Even in M9Glucose minimal medium, in which pyrimidine nucleotides are exclusively synthesized by the de novo biosynthetic pathway, and thus growth is totally dependent on the DHOD activity, VF showed no antimicrobial activity against *E. coli* LF82 (Figure 4B).

### 3.5. In Silico Analysis of Vidofludimus/DHOD Interaction

**Figure 5:**
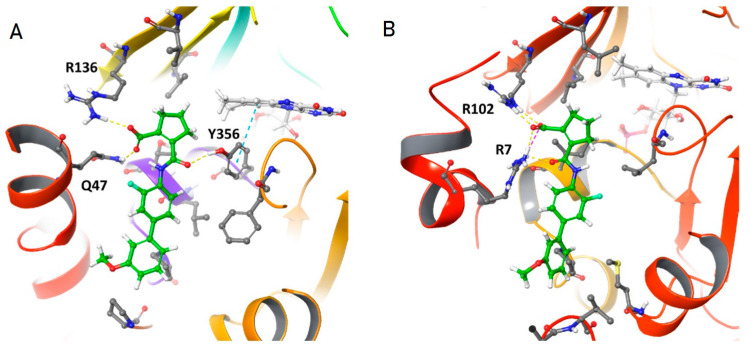
Model of VF-DHOD interaction. (**A**) VF (in green) docked to hDHOD showed an interaction between the VF carboxylic acid moiety and both R136 and Q47. A third interaction is observed between the amide group of VF and the hydroxyl group of Y356. (**B**) When VF is docked to EcDHOD we observed that the VF carboxylic acid moiety form interactions with R102 (equivalent to R136 in human) and R7 (in place of Q47 in human). The VF amide group does not form any interaction.

To further evaluate VF-DHOD interactions, we carried out in silico molecular docking experiments using GLIDE (see Materials and Methods). First we redocked the ligand 5-methoxy-2-[(4-phenoxyphenyl)amino]benzoic acid (R2C) to the corresponding experimentally-determined structure (PDB ID: 2PRL). The ligand shows a GLIDE score of −9.6, with a binding pose identical to the experimental one, where the main interaction is between the carboxylic acid of R2C and R136 and Q47 along with many other polar and non-polar interactions. Then, we docked VF to both human and *E. coli* DHOD (hDHOD and EcDHOD, respectively). Docking on hDHOD showed a GLIDE score of −9.4 with a binding pose comparable to R2C. In particular, the interaction between VF carboxylic acid and R136 and Q47 is maintained (Figure 5A).

Furthermore, a third interaction is formed between the same carboxylic moiety with the hydroxyl group of Y356. Docking to EcDHOD again showed a comparable binding pose (Figure 5B). The carboxylic moiety of VF can interact with a conserved R102 (R136 in human) and with R7 (P44 in human) while lacking a third interaction present in the former. In addition, polar and non-polar interactions are also less optimised, resulting in a reduced GLIDE score of −8. Of note, EcDHOD and hDHOD are structurally similar with an RMSD of less than 1 Å and a sequence identity of 41%. Based on these results, we expect VF to be able to interact with both hDHOD and EcDHOD with similar binding affinities, which would be consistent with the hypothesis that the lack of VF antimicrobial activity might be due to its inability to enter bacterial cells. In contrast, a structural comparison between hDHOD, EcDHOD and the structures of the two DHOD isoenzymes in the Gram positive bacterium *Lactococcus lactis* (the *pyrD* homodimer and the *pyrD-pyrK* heterotetramer, 2DOR and 1EP2, respectively) show that in either DHOD form from this bacterium, the predicted binding site for VF is missing due to the lack of the two N-terminal helices necessary for DHOD-VF interaction (Figure S4). 

## 4. Discussion

The ability of intracellular pathogenic bacteria such as AIEC to invade the gut epithelium and to survive in macrophages is a major trigger for chronic gut inflammation in CD. Thus, specific inhibition of AIEC virulence could be a promising strategy in CD treatment, as it might counteract inflammation without exacerbating gut dysbiosis, which can instead be observed in response to antibiotic therapy [26].

In order to identify potential targets for anti-virulence agents, we have generated a transposon insertion library in the AIEC strain LF82 and looked for mutants unable to survive in a medium mimicking the macrophage environment. Through this approach, we selected an insertion mutation in the *pyrD* gene (LF82*pyrD::*Tn5 mutant), encoding for DHOD, an enzyme part of the de novo pyrimidine biosynthetic pathway. The selection of the *pyrD* mutant is likely due to the lack of exogenous pyrimidines in the medium used in our screening (Figure 1); however, it confirms previous results showing that mutants in AIEC, as well as in *Salmonella*, impaired in their de novo pyrimidine biosynthetic pathway, are indeed unable to survive in macrophages [35,44], which is in line with the more general notion that, in several intracellular pathogens, auxotrophic mutants, either for nucleotides or for some amino acids, are avirulent, mostly because of their inability to grow or survive [45,46,47].

The pleiotropic effects of mutations in the pyrimidine biosynthetic pathway, such as the inability to form biofilm or to produce virulence factors, have already been described in the MG1655 laboratory strain of *E. coli* [36] as well as in other bacteria, such as *P. aeruginosa* [32], possibly via sensing of intracellular pyrimidine nucleotide pools by global regulators [48]. In this work, we showed that, in LF82, inactivation of the *pyrD* gene resulted in the inhibition of surface attachment (Figure 2) via downregulation of genes encoding curli fibers (Figure 2 and Figure 3) and type 1 fimbriae (Figure 3) which are, arguably, the two main proteinaceous adhesion factors in *E. coli*. We had already described that, in *E coli* MG1655, mutations in the pyrimidine biosynthetic genes strongly affected transcription of the *csgDEFG* operon [36], which includes the CsgD regulatory protein presiding to curli and cellulose production; in contrast, in the LF82 background, gene expression downregulation by *pyrD* inactivation was only observed for the *csgBAC* operon, encoding curli structural subunits. This observation would suggest that, in LF82, sensing the lack of intracellular pyrimidines might be more selectively relayed towards turning off curli production, rather than to a more general effect on the CsgD regulon (Figure 3). Inhibition of curli production might be clinically relevant as, although curli production is turned off at 37 °C in laboratory conditions, antibodies against these structures have been isolated in the context of various diseases, suggesting their production would indeed take place during infection. Indeed, curli binding to the TLR1/2 Toll-like receptors and activation of the NOD-like receptor protein 3 inflammasome are thought to contribute to overall inflammation in CD [49,50]. 

While the role of curli fibers in AIEC infection has not yet been fully characterised, type 1 fimbriae are considered *bona fide* AIEC virulence factors that are able to promote bacterial adhesion to epithelial cells [18]. Interestingly, the expression of both curli fibers- and type 1 pili-encoding genes are negatively regulated in the presence of glucose in LF82, suggesting positive control by the CAP master regulator [25], whose activity appears to respond also to pyrimidine intracellular concentrations, as recently proposed [51]. At any rate, the inhibition of pyrimidine biosynthesis appears to have a very extensive impact on AIEC adhesion factors, reiterating its potential as a target for antimicrobial drugs.

Finally, in addition to survival in macrophages, biofilm formation and curli production, as well as cellular motility, another virulence factor in AIEC, was also affected by *pyrD* inactivation, probably via regulation of flagellar motility, as transcription of the *fliC* gene, part of the main flagellar operon, was unaffected in the LF82*pyrD::*Tn5 mutant strain (Figure 3). Unlike survival in the macrophage-mimicking medium and adhesion factors’ production, flagellar motility was not fully restored by uracil supplementation (Figure 2C), possibly suggesting that accumulation of early intermediates of the de novo pyrimidine biosynthesis, rather than pyrimidine availability, might be involved in this process. Indeed, pyrimidine biosynthesis intermediates such as N-carbamoyl-aspartate can modulate production of c-di-GMP, a signal molecule involved in several processes, including flagellar motility [52].

The pleiotropic effects of the *pyrD* mutation on several virulence-related processes would make DHOD, the product of the *pyrD* gene, a suitable target for novel inhibitors of AIEC growth and/or virulence. This led us to hypothesize that VF, a known inhibitor of human DHOD studied in clinical trials as an anti-inflammatory drug in the treatment of CD [43,53], could possess antimicrobial and antivirulence activity against AIEC via bacterial DHOD inhibition. Indeed, antimicrobial activity against microorganisms able to trigger chronic inflammation in CD has been proposed to be at least partly responsible for the anti-inflammatory activity of mercaptopurines, which, like VF, can inhibit de novo nucleotide biosynthesis in bacteria [24,25,54]. However, unlike mercaptopurines, VF possesses little or no activity either against AIEC or Gram positive microorganisms (Figure 4), although molecular docking analysis (Figure 5) predicts that VF can bind human and *E. coli* DHOD with similar affinity. The lack of antimicrobial activity by VF against AIEC (Figure 4) might thus depend on VF poor penetration into the cell, despite its low molecular mass (355.12), a behaviour observed also for several antimicrobial agents, such as clindamycin (MW 424.98) [55]. In contrast, comparison of the *E. coli* DHOD structure to both DHOD isoenzymes of the Gram positive, probiotic bacterium *Lactococcus lactis* (Figure S4) shows the absence of any potential binding site for VF in either DHOD isoform. Our results reiterate previous observations on the functional diversity of DHOD among bacteria [56] which, in addition to known structural differences between human and *E. coli* DHOD [57], would suggest the possibility to design DHOD inhibitors that might specifically target only a subset of selected bacterial species. In the context of a chronic inflammatory disease such as CD, targeting *E. coli* DHOD without affecting beneficial bacteria in the gut microbiota could eradicate an important trigger for chronic inflammation and help restore gut microbiota eubiosis, thus representing a promising therapeutic strategy.

## Data Availability

All data generated or analyzed during this study are included in this published article (and its supplementary information files).

## Supplementary Materials

The following are available online at http://www.mdpi.com/article/10.3390/microorganisms10030537/s1, Table S1: List of oligonucleotides used for qRT-PCR experiments. Figure S1: Growth rates and lag phases of LF82 wild type strain and of the LF82*pyrD::*Tn5 mutant in Acid medium supplemented either with DMSO or uracil. Figure S2: Optical density of overnight cultures of LF82 wild type strain and of the LF82*pyrD::*Tn5 mutant in YESCA medium in microtiter plates used for biofilm determination experiments. Figure S3: (A) Viability of the LF82*pyrD::*Tn5 mutant compared to its parental strain on LB Agar and MacConkey media. (B) Relative expression levels of the *lptD* gene. Figure S4: structures of *Lactococcus lactis* PyrD protein.

## References

[1] de Souza H.S.P., Fiocchi C. (2016). Immunopathogenesis of IBD: Current State of the Art. Nat. Rev. Gastroenterol..

[2] Duerr R.H., Taylor K.D., Brant S.R., Rioux J.D., Silverberg M.S., Daly M.J., Steinhart A.H., Abraham C., Regueiro M., Griffiths A. (2006). A Genome-Wide Association Study Identifies IL23R as an Inflammatory Bowel Disease Gene. Science.

[3] Villani A.-C., Lemire M., Fortin G., Louis E., Silverberg M.S., Collette C., Baba N., Libioulle C., Belaiche J., Bitton A. (2009). Common Variants in the NLRP3 Region Contribute to Crohn’s Disease Susceptibility. Nat. Genet..

[4] Ng S.C., Bernstein C.N., Vatn M.H., Lakatos P.L., Loftus E.V., Tysk C., O’Morain C., Moum B., Colombel J.-F., on behalf of the Epidemiology and Natural History Task Force of the International Organization of Inflammatory Bowel Disease (IOIBD) (2013). Geographical Variability and Environmental Risk Factors in Inflammatory Bowel Disease. Gut.

[5] Wang K., Wu L., Dou C., Guan X., Wu H., Liu H. (2016). Research Advance in Intestinal Mucosal Barrier and Pathogenesis of Crohn’s Disease. Gastroenterol. Res. Pract..

[6] Geremia A., Biancheri P., Allan P., Corazza G.R., Sabatino A.D. (2014). Innate and Adaptive Immunity in Inflammatory Bowel Disease. Autoimmun. Rev..

[7] Ahmed I., Roy B.C., Khan S.A., Septer S., Umar S. (2016). Microbiome, Metabolome and Inflammatory Bowel Disease. Microorganisms.

[8] Nishida A., Inoue R., Inatomi O., Bamba S., Naito Y., Andoh A. (2018). Gut Microbiota in the Pathogenesis of Inflammatory Bowel Disease. Clin. J. Gastroenterol..

[9] Walters W.A., Xu Z., Knight R. (2014). Meta-analyses of Human Gut Microbes Associated with Obesity and IBD. FEBS Lett..

[10] Gevers D., Kugathasan S., Knights D., Kostic A.D., Knight R., Xavier R.J. (2017). A Microbiome Foundation for the Study of Crohn’s Disease. Cell Host Microbe.

[11] Mentella M.C., Scaldaferri F., Pizzoferrato M., Gasbarrini A., Miggiano G.A.D. (2020). Nutrition, IBD and Gut Microbiota: A Review. Nutrients.

[12] Darfeuille-Michaud A., Boudeau J., Bulois P., Neut C., Glasser A.-L., Barnich N., Bringer M.-A., Swidsinski A., Beaugerie L., Colombel J.-F. (2004). High Prevalence of Adherent-Invasive *Escherichia coli* Associated with Ileal Mucosa in Crohn’s Disease. Gastroenterology.

[13] Schippa S., Conte M.P., Borrelli O., Iebba V., Aleandri M., Seganti L., Longhi C., Chiarini F., Osborn J., Cucchiara S. (2009). Dominant Genotypes in Mucosa-Associated *Escherichia coli* Strains from Pediatric Patients with Inflammatory Bowel Disease. Inflamm. Bowel Dis..

[14] Thomazini C.M., Samegima D.A.G., Rodrigues M.A.M., Victoria C.R., Rodrigues J. (2011). High Prevalence of Aggregative Adherent *Escherichia coli* Strains in the Mucosa-Associated Microbiota of Patients with Inflammatory Bowel Diseases. Int. J. Med. Microbiol..

[15] Glasser A.-L., Boudeau J., Barnich N., Perruchot M.-H., Colombel J.-F., Darfeuille-Michaud A. (2001). Adherent Invasive *Escherichia coli* Strains from Patients with Crohn’s Disease Survive and Replicate within Macrophages without Inducing Host Cell Death. Infect. Immun..

[16] Eaves-Pyles T., Allen C.A., Taormina J., Swidsinski A., Tutt C.B., Jezek G.E., Islas-Islas M., Torres A.G. (2008). *Escherichia coli* Isolated from a Crohn’s Disease Patient Adheres, Invades, and Induces Inflammatory Responses in Polarized Intestinal Epithelial Cells. Int. J. Med. Microbiol..

[17] Rolhion N., Darfeuille-Michaud A. (2007). Adherent-Invasive *Escherichia coli* in Inflammatory Bowel Disease. Inflamm. Bowel Dis..

[18] Boudeau J., Barnich N., Darfeuille-Michaud A. (2001). Type 1 Pili-mediated Adherence of *Escherichia coli* Strain LF82 Isolated from Crohn’s Disease Is Involved in Bacterial Invasion of Intestinal Epithelial Cells. Mol. Microbiol..

[19] Barnich N., Boudeau J., Claret L., Darfeuille-Michaud A. (2003). Regulatory and Functional Co-operation of Flagella and Type 1 Pili in Adhesive and Invasive Abilities of AIEC Strain LF82 Isolated from a Patient with Crohn’s Disease. Mol. Microbiol..

[20] Cieza R.J., Hu J., Ross B.N., Sbrana E., Torres A.G. (2015). The IbeA Invasin of Adherent-Invasive *Escherichia coli* Mediates Interaction with Intestinal Epithelia and Macrophages. Infect. Immun..

[21] Chassaing B., Darfeuille-Michaud A. (2013). The σ^E^ Pathway Is Involved in Biofilm Formation by Crohn’s Disease-Associated Adherent-Invasive *Escherichia coli*. J. Bacteriol..

[22] Fanelli G., Pasqua M., Colonna B., Prosseda G., Grossi M. (2020). Expression Profile of Multidrug Resistance Efflux Pumps During Intracellular Life of Adherent-Invasive *Escherichia coli* Strain LF82. Front. Microbiol..

[23] D’Haens G.R., Vermeire S., Assche G.V., Noman M., Aerden I., Olmen G.V., Rutgeerts P. (2008). Therapy of Metronidazole With Azathioprine to Prevent Postoperative Recurrence of Crohn’s Disease: A Controlled Randomized Trial. Gastroenterology.

[24] Shin S.J., Collins M.T. (2008). Thiopurine Drugs Azathioprine and 6-Mercaptopurine Inhibit *Mycobacterium paratuberculosis* Growth In Vitro. Antimicrob. Agents Chemother..

[25] Migliore F., Macchi R., Landini P., Paroni M. (2018). Phagocytosis and Epithelial Cell Invasion by Crohn’s Disease-Associated Adherent-Invasive *Escherichia coli* Are Inhibited by the Anti-Inflammatory Drug 6-Mercaptopurine. Front. Microbiol..

[26] Nitzan O., Elias M., Peretz A., Saliba W. (2016). Role of Antibiotics for Treatment of Inflammatory Bowel Disease. World J. Gastroenterol..

[27] Ganji-Arjenaki M., Rafieian-Kopaei M. (2018). Probiotics Are a Good Choice in Remission of Inflammatory Bowel Diseases: A Meta Analysis and Systematic Review. J. Cell Physiol..

[28] Ghouri Y.A., Richards D.M., Rahimi E.F., Krill J.T., Jelinek K.A., DuPont A.W. (2014). Systematic Review of Randomized Controlled Trials of Probiotics, Prebiotics, and Synbiotics in Inflammatory Bowel Disease. Clin. Exp. Gastroenterol..

[29] Butterworth A.D., Thomas A.G., Akobeng A.K. (2008). Probiotics for Induction of Remission in Crohn’s Disease. Cochrane Database Syst. Rev..

[30] Leccese G., Bibi A., Mazza S., Facciotti F., Caprioli F., Landini P., Paroni M. (2020). Probiotic *Lactobacillus* and *Bifidobacterium* Strains Counteract Adherent-Invasive *Escherichia coli* (AIEC) Virulence and Hamper IL-23/Th17 Axis in Ulcerative Colitis, but Not in Crohn’s Disease. Cells.

[31] Durand J.M.B., Björk G.R. (2009). Metabolic Control through Ornithine and Uracil of Epithelial Cell Invasion by *Shigella flexneri*. Microbiology.

[32] Ueda A., Attila C., Whiteley M., Wood T.K. (2009). Uracil Influences Quorum Sensing and Biofilm Formation in *Pseudomonas aeruginosa* and Fluorouracil Is an Antagonist. Microb. Biotechnol..

[33] Boudeau J., Glasser A.-L., Masseret E., Joly B., Darfeuille-Michaud A. (1999). Invasive Ability of an *Escherichia coli* Strain Isolated from the Ileal Mucosa of a Patient with Crohn’s Disease. Infect. Immun..

[34] Bringer M.-A., Rolhion N., Glasser A.-L., Darfeuille-Michaud A. (2007). The Oxidoreductase DsbA Plays a Key Role in the Ability of the Crohn’s Disease-Associated Adherent-Invasive *Escherichia coli* Strain LF82 To Resist Macrophage Killing. J. Bacteriol..

[35] Thompson A.P., O’Neill I., Smith E.J., Catchpole J., Fagan A., Burgess K.E.V., Carmody R.J., Clarke D.J. (2016). Glycolysis and Pyrimidine Biosynthesis Are Required for Replication of Adherent–Invasive *Escherichia coli* in Macrophages. Microbiology.

[36] Garavaglia M., Rossi E., Landini P. (2012). The Pyrimidine Nucleotide Biosynthetic Pathway Modulates Production of Biofilm Determinants in *Escherichia coli*. PLoS ONE.

[37] Genevaux P., Bauda P., DuBow M.S., Oudega B. (1999). Identification of Tn 10 Insertions in the RfaG, RfaP, and GalU Genes Involved in Lipopolysaccharide Core Biosynthesis That Affect *Escherichia coli* Adhesion. Arch. Microbiol..

[38] Møller A.K., Leatham M.P., Conway T., Nuijten P.J.M., de Haan L.A., Krogfelt K.A., Cohen P.S. (2003). An *Escherichia coli* MG1655 Lipopolysaccharide Deep-Rough Core Mutant Grows and Survives in Mouse Cecal Mucus but Fails To Colonize the Mouse Large Intestine. Infect. Immun..

[39] Dartigalongue C., Missiakas D., Raina S. (2001). Characterization of the *Escherichia coli* σ^E^ Regulon. J. Biol. Chem..

[40] Tam C., Missiakas D. (2005). Changes in Lipopolysaccharide Structure Induce the σ^E^-dependent Response of *Escherichia coli*. Mol. Microbiol..

[41] Römling U., Rohde M., Olsén A., Normark S., Reinköster J. (2000). AgfD, the Checkpoint of Multicellular and Aggregative Behaviour in *Salmonella typhimurium* Regulates at Least Two Independent Pathways. Mol. Microbiol..

[42] Zogaj X., Nimtz M., Rohde M., Bokranz W., Römling U. (2001). The Multicellular Morphotypes of *Salmonella typhimurium* and *Escherichia coli* Produce Cellulose as the Second Component of the Extracellular Matrix. Mol. Microbiol..

[43] Herrlinger K.R., Diculescu M., Fellermann K., Hartmann H., Howaldt S., Nikolov R., Petrov A., Reindl W., Otte J.M., Stoynov S. (2013). Efficacy, Safety and Tolerability of Vidofludimus in Patients with Inflammatory Bowel Disease: The ENTRANCE Study. J. Crohn’s Colitis.

[44] Ellis M.J., Tsai C.N., Johnson J.W., French S., Elhenawy W., Porwollik S., Andrews-Polymenis H., McClelland M., Magolan J., Coombes B.K. (2019). A Macrophage-Based Screen Identifies Antibacterial Compounds Selective for Intracellular *Salmonella typhimurium*. Nat. Commun..

[45] Bange F.C., Brown A.M., Jacobs W.R. (1996). Leucine Auxotrophy Restricts Growth of *Mycobacterium bovis* BCG in Macrophages. Infect. Immun..

[46] Pilatz S., Breitbach K., Hein N., Fehlhaber B., Schulze J., Brenneke B., Eberl L., Steinmetz I. (2006). Identification of *Burkholderia pseudomallei* Genes Required for the Intracellular Life Cycle and In Vivo Virulence. Infect. Immun..

[47] Smith D.A., Parish T., Stoker N.G., Bancroft G.J. (2001). Characterization of Auxotrophic Mutants of *Mycobacterium tuberculosis* and Their Potential as Vaccine Candidates. Infect. Immun..

[48] Beaumont H.J.E., Gallie J., Kost C., Ferguson G.C., Rainey P.B. (2009). Experimental Evolution of Bet Hedging. Nature.

[49] Tükel Ç., Nishimori J.H., Wilson R.P., Winter M.G., Keestra A.M., Putten J.P.M.V., Bäumler A.J. (2010). Toll-like Receptors 1 and 2 Cooperatively Mediate Immune Responses to Curli, a Common Amyloid from Enterobacterial Biofilms. Cell Microbiol..

[50] Rapsinski G.J., Wynosky-Dolfi M.A., Oppong G.O., Tursi S.A., Wilson R.P., Brodsky I.E., Tükel Ç. (2014). Toll-like Receptor 2 and NLRP3 Cooperate to Recognize a Functional Bacterial Amyloid, Curli. Infect. Immun..

[51] Lauritsen I., Frendorf P.O., Capucci S., Heyde S.A.H., Blomquist S.D., Wendel S., Fischer E.C., Sekowska A., Danchin A., Nørholm M.H.H. (2021). Temporal Evolution of Master Regulator Crp Identifies Pyrimidines as Catabolite Modulator Factors. Nat. Commun..

[52] Rossi E., Motta S., Aliverti A., Cossu F., Gourlay L., Mauri P., Landini P. (2017). Cellulose Production Is Coupled to Sensing of the Pyrimidine Biosynthetic Pathway via c-di-GMP Production by the DgcQ Protein of *Escherichia coli*. Environ. Microbiol..

[53] Muehler A., Kohlhof H., Groeppel M., Vitt D. (2020). Safety, Tolerability and Pharmacokinetics of Vidofludimus Calcium (IMU-838) After Single and Multiple Ascending Oral Doses in Healthy Male Subjects. Eur. J. Drug Metab. Pharmacokinet..

[54] Antoniani D., Rossi E., Rinaldo S., Bocci P., Lolicato M., Paiardini A., Raffaelli N., Cutruzzolà F., Landini P. (2013). The Immunosuppressive Drug Azathioprine Inhibits Biosynthesis of the Bacterial Signal Molecule Cyclic-Di-GMP by Interfering with Intracellular Nucleotide Pool Availability. Appl. Microbiol. Biot..

[55] Leclercq R., Courvalin P. (1991). Intrinsic and Unusual Resistance to Macrolide, Lincosamide, and Streptogramin Antibiotics in Bacteria. Antimicrob. Agents Chemother..

[56] Nørager S., Jensen K.F., Björnberg O., Larsen S. (2002). *E. Coli* Dihydroorotate Dehydrogenase Reveals Structural and Functional Distinctions between Different Classes of Dihydroorotate Dehydrogenases. Structure.

[57] Marcinkeviciene J., Rogers M.J., Kopcho L., Jiang W., Wang K., Murphy D.J., Lippy J., Link S., Chung T.D.Y., Hobbs F. (2000). Selective Inhibition of Bacterial Dihydroorotate Dehydrogenases by Thiadiazolidinediones. Biochem. Pharmacol..

