# Supplementary material for: Inactivation of the Pyrimidine Biosynthesis pyrD Gene Negatively Affects Biofilm Formation and Virulence Determinants in the Crohn’s Disease-Associated Adherent Invasive Escherichia coli LF82 Strain
Source: Microorganisms. 2022 Feb 28;10(3):537. doi: 10.3390/microorganisms10030537 (PMC8956108; doi:10.3390/microorganisms10030537)
Supplement: Supplementary file 1 [file microorganisms-10-00537-s001.zip › microorganisms-1589884-supplementary.pdf]

**Supplementary Materials**

**Table S1**. Oligonucleotides used for RT-PCR experiments.

| Name | Sequence (5′ → 3′) |
|---|---|
| 16s_RT_for | TGTCGTCAGCTCGTGTCGTGA |
| 16s_RT_rev | ATCCCCACCTTCCTCCGGT |
| csgB_RT_for | CATAATTGGTCAAGCTGGGACTAA |
| csgB_RT_rev | GCAACAACCGCCAAAAGTTT |
| csgD_RT_for | CCCGTACCGCGACATTG |
| csgD_RT_rev | CGTTCTTGATCCTCCATGG |
| bcsA_RT_for | TCGCGATTATCGTCGTCACG |
| bcsA_RT_rev | GGGTGCTCCAGCGGAATAAA |
| adrA_RT_for | GGCTGGGTCAGCTACCAG |
| adrA_RT_rev | CGTCGGTTATACACGCCCG |
| fimA_RT_for | CGCTTGCGCAGTTGATGCAG |
| fimA_RT_rev | CCGTCCCCAAGAAGGCAACA |
| fliC_RT_for | CAACTTACAGCGTATCCGTG |
| fliC_RT_rev | CGTTCACGCCGTTGAACTG |
| lptD_RT_for | GATGCGCTCGGTAATGTCC |
| lptD_RT_rev | CACCTTCCCAGACGTTGGT |

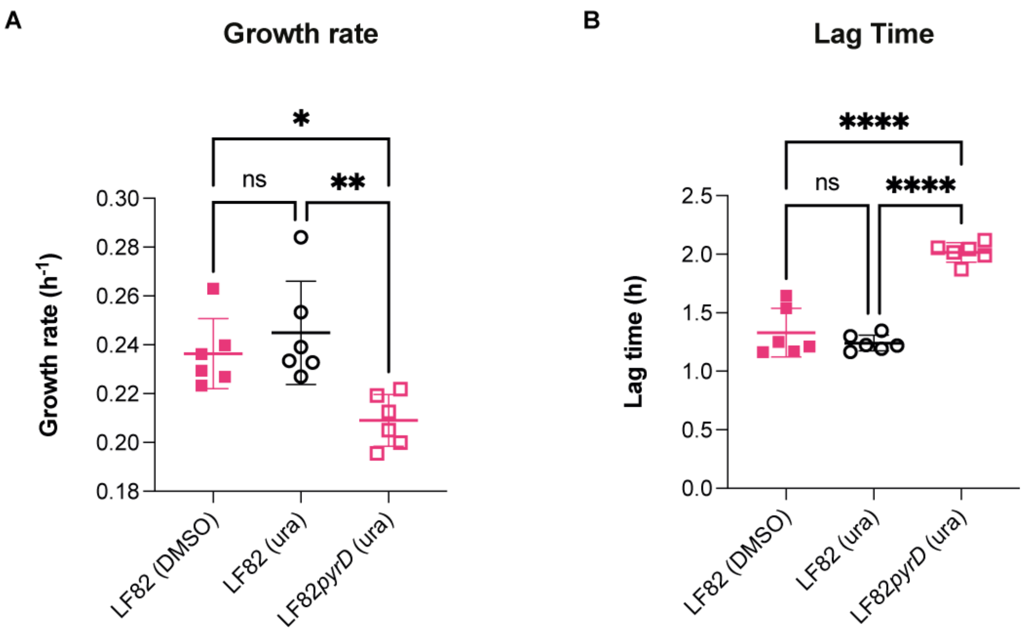


**Figure S1**. Analysis of growth rates (**A**) and lag time after inoculation (**B**) in Acid Medium for LF82 grown either with 0.25% DMSO, or 0.25mM uracil in DMSO, and for LF82*pyrD*::Tn5 in the presence of 0.25mM uracil. Results of six independent experiments are shown. *, *p*-value < 0.05; **, *p*-value < 0.01; ****, *p*-value < 0.0001, one-way ANOVA with Tukey's test for multiple comparisons.

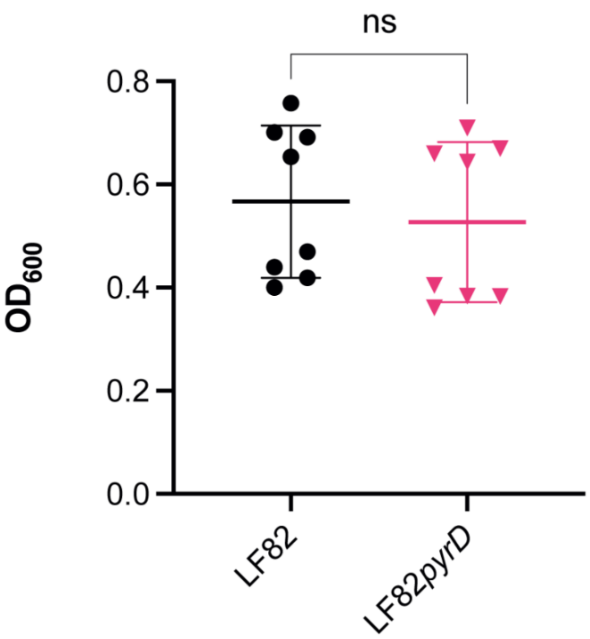


**Figure S2**. Optical density of overnight cultures of LF82 and LF82*pyrD*::Tn5 grown in YESCA medium in microtiter plates at 30 °C (conditions used for biofilm determination with crystal violet shown in Figure 2). Each point represents an independent biological replicate. ns, not significant (*p*-value = 0.89), Student t test.

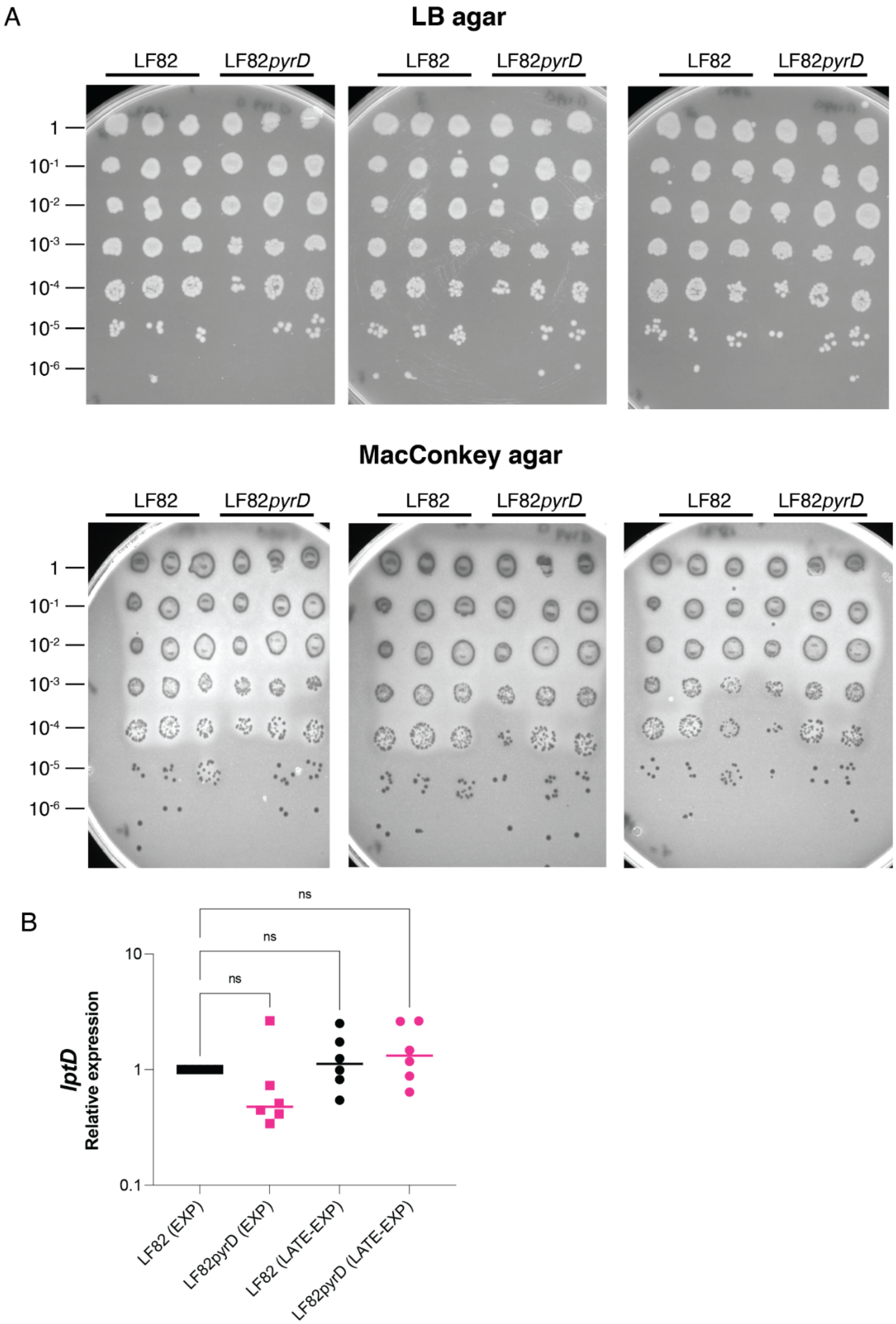


**Figure S3**. (**A**) Plating efficiency of LF82 and its LF82*pyrD*::Tn5 mutant derivative on Luria Agar (LA) and MacConkey media. Overnight cultures grown in LB medium were adjusted to OD600nm=1.0 and serially diluted 1:10 in microtiter plates to a final dilution of $10^{-6}$. Dilutions were replicated on both LA and MacConkey. (**B**) Determination of gene expression levels in the LF82 vs the LF82*pyrD*::Tn5 (LF82*pyrD*) strain by qRT-PCR. Results of six independent experiments are shown. ns, not significant, one-way ANOVA with Tukey's test for multiple comparisons.

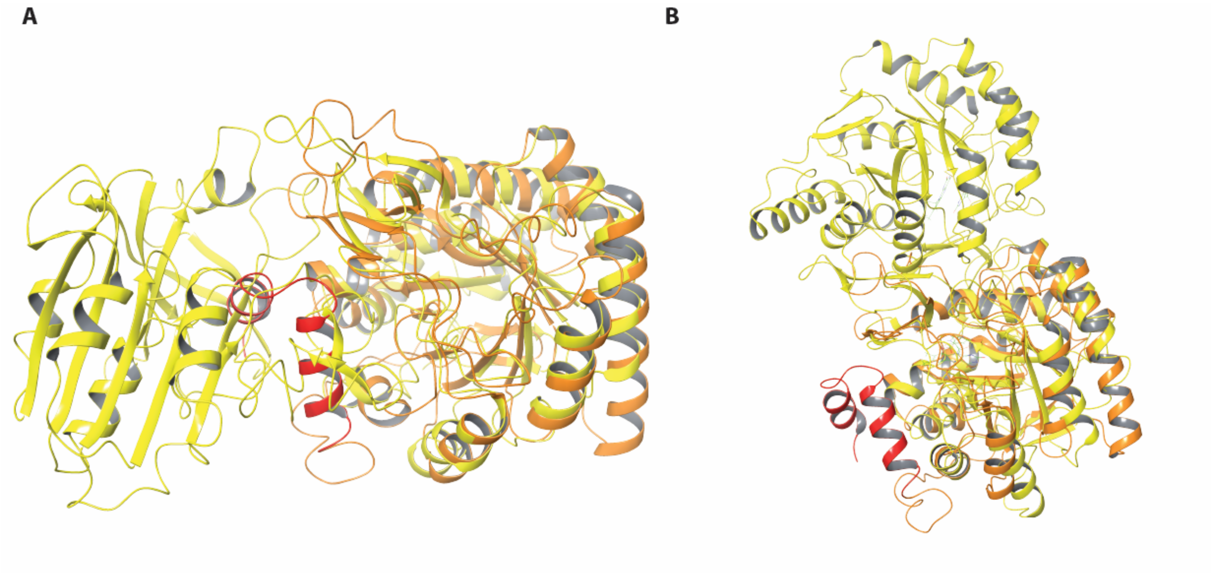


**Figure S4. (A**) Crystal structure of *Lactococcus lactis* DHOD type B (pdb 1EP2) where PyrD domain is superimposed to the crystal structure of EcDHOD (pdb 1F76). (**B**) Crystal structure of *Lactococcus lactis* DHOD type A (pdb 2DOR) where one of the PyrD domains is superimposed to with the crystal structure of EcDHOD (pdb 1F76).
